# Supplementary material for: Preserving integrity: innovative in vitro methods for extracellular matrix decellularization and collagen purification
Source: J Biol Eng. 2026 Jan 23;20:35. doi: 10.1186/s13036-025-00617-6 (PMC12910738; doi:10.1186/s13036-025-00617-6)
Supplement: Supplementary file 1 — Supplementary Material 1 [file 13036_2025_617_MOESM1_ESM.docx]

**SUPPLEMENTARY FIGURE.**

**Fig. S1. ¹³C CP/MAS ssNMR spectra of unlabelled/natural abundance FSOB ECM following collagen isolation steps.** Collagen isolation steps included either freeze-thaw cell lysis or Triton X-100 cell lysis ("detergent"), followed by chymotrypsin digestion. The control spectrum was represented by commercially available bovine collagen type I. Only after the chymotrypsin digestion step, collagen from the ECM was clean enough to correlate well with that of commercial collagen. Samples were freeze-dried and spectra were recorded on a 400 MHz spectrometer at room temperature using a 10 kHz MAS and the following number of scans: 32k (chymotrypsin), 8k (both freeze-thaw and detergent) and 256 (commercial collagen).

| **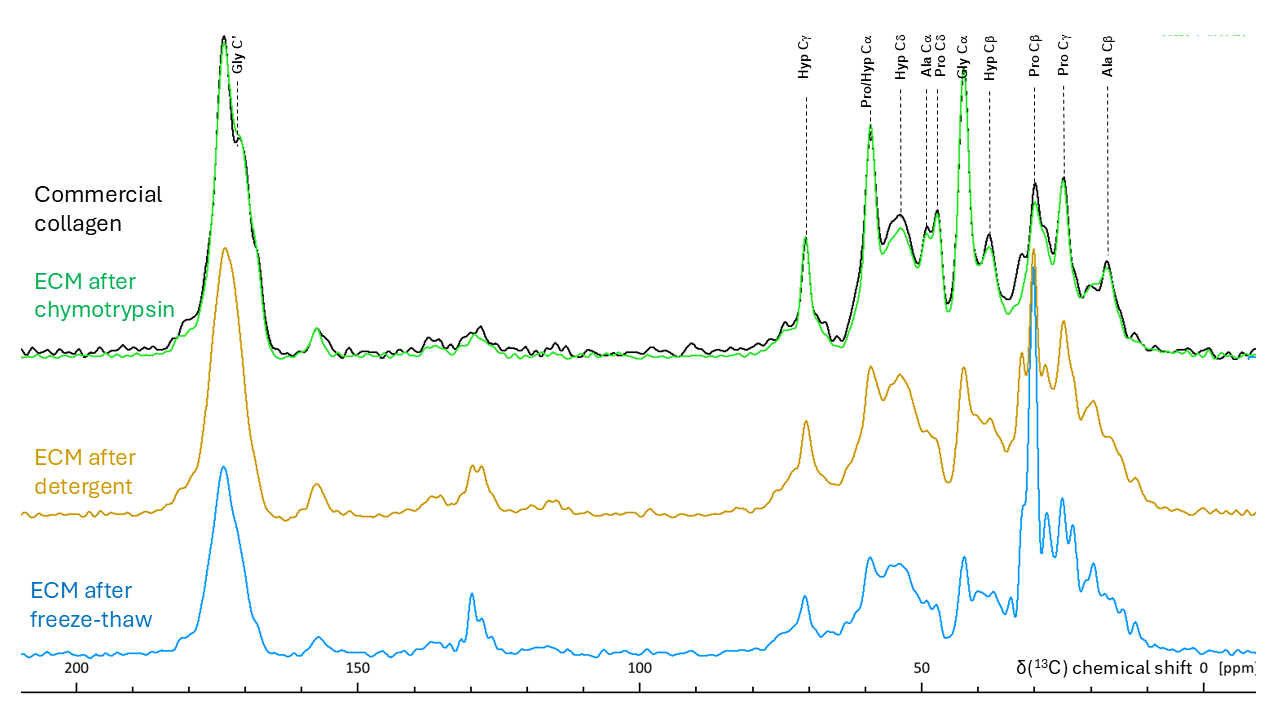** |
| --- |
